# Prophages in marine *Citromicrobium*: diversity, activity, and interaction with the host

**DOI:** 10.1093/ismeco/ycaf148

**Published:** 2025-08-29

**Authors:** Ruijie Ma, Bu Xu, Xiaowei Chen, Qiang Sun, Yingying Li, Qiang Zheng, Nianzhi Jiao, Rui Zhang

**Affiliations:** College of Civil and Transportation Engineering, Shenzhen University, Shenzhen, Guangdong, 518060, China; Archaeal Biology Center, Synthetic Biology Research Center, Shenzhen Key Laboratory of Marine Microbiome Engineering, Key Laboratory of Marine Microbiome Engineering of Guangdong Higher Education Institutes, Institute for Advanced Study, Shenzhen University, Shenzhen, Guangdong, 518060, China; College of Civil and Transportation Engineering, Shenzhen University, Shenzhen, Guangdong, 518060, China; Archaeal Biology Center, Synthetic Biology Research Center, Shenzhen Key Laboratory of Marine Microbiome Engineering, Key Laboratory of Marine Microbiome Engineering of Guangdong Higher Education Institutes, Institute for Advanced Study, Shenzhen University, Shenzhen, Guangdong, 518060, China; State Key Laboratory of Marine Environmental Science, College of Ocean and Earth Sciences, Institute of Marine Microbes and Ecospheres, Xiamen University, Xiamen, Fujian, 361102, China; Department of Infection Prevention and Control, The 964th Hospital, Changchun, Jilin, 130021, China; State Key Laboratory of Marine Environmental Science, College of Ocean and Earth Sciences, Institute of Marine Microbes and Ecospheres, Xiamen University, Xiamen, Fujian, 361102, China; State Key Laboratory of Marine Environmental Science, College of Ocean and Earth Sciences, Institute of Marine Microbes and Ecospheres, Xiamen University, Xiamen, Fujian, 361102, China; State Key Laboratory of Marine Environmental Science, College of Ocean and Earth Sciences, Institute of Marine Microbes and Ecospheres, Xiamen University, Xiamen, Fujian, 361102, China; Archaeal Biology Center, Synthetic Biology Research Center, Shenzhen Key Laboratory of Marine Microbiome Engineering, Key Laboratory of Marine Microbiome Engineering of Guangdong Higher Education Institutes, Institute for Advanced Study, Shenzhen University, Shenzhen, Guangdong, 518060, China

**Keywords:** prophage, Mu-like phages, polylysogeny, piggyback-the-winner, prophage within-host competition

## Abstract

Lysogeny was frequently detected in marine ecosystems, while how temperate phage genomes (prophages) impact marine microbial population or individual dynamics remained poorly understood. Using marine *Citromicrobium* strain collection as a model system, we revealed that 58% (22/38) were lysogens harboring 31 prophages that can be grouped into five novel genera (φA–φE). Prophage-encoded genes constituted 9% of host accessory genome, significantly expanding the microdiversity among citromicrobial clonal strains. Metagenomic abundance correlations indirectly supported the “Piggyback-the-Winner” dynamics for φA/φE, evidenced by their sublinear growth pattern with increasing host abundance. Most prophages were capable of spontaneous induction and exhibited high lytic activity when triggered by mitomycin C. Importantly, host-range profiling revealed these prophages deployed a dual “Kill-the-Relatives” and “Colonize-the-Relatives” strategy, and meanwhile, they protected parental host strains through superinfection immunity and enhanced phage resistance with greater prophage carriage. Sequencing data showed the dominance of Mu-like phages over non-Mu-like partners upon induced from the double lysogens. Our analysis further hinted at a unique Mu-type within-host competitive strategy: selectively targeting genes of co-resident prophages and host hypothetical genes, while avoiding self-damage and host metabolic genes potentially essential for phage lytic growth or progeny release. Collectively, this work establishes prophages as key architects of bacterial adaptation and provides new perspectives for prophage-driven evolution in marine bacterial hosts.

## Introduction

Bacteriophages are the most abundant biological entities in marine ecosystems, surpassing prokaryotic populations by approximately an order of magnitude [1]. Phage lytic infections account for 20%–40% of daily marine bacterial mortality [2], significantly redirecting carbon flow from higher trophic levels into microbial recycling processes [3]. Meanwhile, phages are crucial agents for horizontal gene transfer (HGT) among bacterial hosts and co-infecting phages, constituting the largest reservoir of unexplored genetic diversity in marine environments [3]. The ecological and biogeochemical consequences of phage–bacterium interactions were fundamentally shaped by phage infection strategies, particularly either lytic or lysogenic infections. While lytic infections, characterized by rapid phage propagation and host cell lysis, have been extensively studied, temperate phages capable of establishing stable symbiosis (lysogeny) with its host bacterium have received less attention. Lysogeny enables temperate phages to maintain their genomes as prophages within host chromosome, replicating passively with host cell division until specific environmental cues trigger lytic induction [4].

Research on lysogeny in marine ecosystems initiated only in the late 20th century and has progressed more slowly than comparable studies in medical and food microbiology model systems, such as *Escherichia*, *Pseudomonas*, and *Vibrio*. Field investigations have revealed that lysogeny prevalence exhibits significant spatial and temporal variation, fluctuating with seasonal patterns [5, 6], oceanic regions [7, 8], and specific host microbial taxa [9]. The development of high-throughput sequencing technologies, coupled with bioinformatic pipelines for prophage prediction based on signature genes (e.g. integrases and repressors), has enabled large-scale identification of temperate phage genomes from metagenomic data [10–12]. These advances have established a consensus that ~50% of marine bacteria harbor prophages [13]. Two competing frameworks dominate current understanding of phage-host population dynamics: the “Kill-the-winner” model [14] posits rapidly growing hosts are more likely targeted by lytic phages, while the “Piggyback-the-Winner” (PtW) [15] model links high host density to phage lysogeny dominance [16, 17]. This theoretical dichotomy, combined with the complexity of marine phage-bacteria interactions, highlights critical gaps in our understanding of how temperate phages shape host bacterial dynamics in oceanic ecosystems.

*Citromicrobium* spp., as members of the Alpha-IV proteobacterial subcluster, are capable of aerobic anoxygenic photosynthesis to capture light energy, representing key players in marine carbon cycle [18]. The great environmental adaptability [19] enabled members of this genus, predominantly *C. bathyomarinum*, have been isolated globally across diverse marine habitats [20, 21]. This study focuses on prophages within this ecologically significant genus, examining their prevalence, diversity, and activity, and to elucidate their potential impacts on host evolutionary and ecological dynamics.

## Materials and methods

### Strain culture, sequencing, and bioinformatic analysis

Citromicrobial cultures were maintained aerobically in rich organic (RO) medium [19] at 28°C with a shaking speed of 160 rpm·min^−1^. Genomic DNA sequencing employed both Illumina MiSeq (short-read) or PacBio RS II (long-read) platforms, with long-read sequencing particularly helpful for resolving similar prophage content in polylysogenic strains, such as MCCC1A09559 (φD1/φD2) and JL2201 (φA3/φB3/φB5). We have deposited thirty-eight citromicrobial genomes in the NCBI GenBank database, and their accession numbers are listed in Supplementary Table 1.

Average nucleotide identities (ANI) of citromicrobial genomes was determined using JSpecies 1.2.1 [22]. Bacterial whole-genome phylogeny was constructed using progressive MAUVE algorithm [23]. To identify bacterial genetic islands, multiple-genome comparisons were conducted and visualized using the BLAST Ring Image Generator (BRIG) [24]. Core/pan-genome analysis of citromicrobial genomes was conducted by Prokka annotation [25] followed by Roary assessment [26]. Single nucleotide polymorphisms (SNPs) were extracted from core genome alignments with insertion sequence-associated false positives removed using Gubbins [27].

### Prophage identification and border curation

Prophage candidates were initially identified through manual inspection of clustered phage-related genes in bacterial annotation files. Two phage identification tools, PHAST [28] and CheckV [29], were employed to supplement manual inspection during the initial screening of prophage candidates. Precise prophage boundaries were determined by comparative BLASTn alignment between parental bacterial genomes and their isogenic prophage-free counterparts, such that flanking host sequences can be precisely excluded. Complete genomic coordinates for all identified prophages were documented in Supplementary Data Set 1.

### Network analysis

To create comparative phage profiles, citromicrobial prophages and 3464 genomes (358 468 proteins) of prokaryotic viruses from RefSeq (version 99) were analyzed using vConTACT2 [30] to construct proteomic similarity networks. Prokaryotic viruses showing similarity scores of <1 with citromicrobial prophages were excluded for subsequent analysis. Network visualization in Cytoscape 3.8.0 [31] employed a spring-embedded layout weighted by vConTACT2 similarity scores, where node proximity reflected shared protein cluster content.

### Prophage bioinformatics analysis

Prophage genes were annotated using BLASTp, Virfam [32], and HHpred [33], with the E-value cutoff of <10^−3^. Phage genome maps were generated using JavaScript. Complete phage amino acid profiles were submitted to the VICTOR server for whole-genome tree construction using the genome BLAST distance phylogeny method under the recommended settings for prokaryotic viruses [34]. The resulting phylogenetic tree was annotated and visualized using the iTOL v6 [35].

### Abundance correlations of citromicrobial prophages and their hosts

To examine the abundance distribution patterns of citromicrobial prophages and their hosts in global marine environments, we analyzed metagenomic datasets from the Tara Oceans expeditions (2009–2013) [36]. Raw metagenomic reads were quality-filtered using fastp [37] with the parameters “—cut_mean_quality 20—detect_adapter_fo_pe”. Prophage regions within host contigs were masked using Bedtools (v. 2.27.1) [38] to avoid mapping mistakes of host contigs from prophage reads. Read mapping was performed using Bowtie2 (v. 2.3.5) [39] with the “—very-sensitive—no-unal” parameters, followed by sorting with SAMtools (v. 1.9) [40]. The mapping results were then filtered using BamM (v. 1.7.3) (https://github.com/minillinim/BamM) with thresholds of 99% identity and 75% read coverage.

Read counts for each contig were generated from the filtered BAM files using bbmap (http://jgi.doe.gov/data-and-tools/bb-tools/). The relative abundance of each citromicrobial prophage and its host was calculated as reads per kb of genome per mbp of each metagenomic sample (RPKM) according to Tully’s method [41], retaining samples where RPKM values exceeded 0.1 for hosts or 0.01 for prophages. For stations containing samples with both cellular and virion fractions, we specifically analyzed abundance correlations between citromicrobial prophages and their hosts. Spearman’s correlation analyses comparing virus abundances, virus-to-host ratios (VHR), and host abundances were conducted using the “rcorr” function of the Hmisc R package (https://cran.r-project.org/web/packages/Hmisc/).

### Prophage induction experiment

Mitomycin C (MmC; Sigma-Aldrich, MO, USA) was used as an inducing agent to activate cellular SOS responses and initiate prophage transition to lytic growth [42]. A 1 L early-exponential-phase bacterial culture was exposed to MmC (final concentration, 0.5 mg·L^−1^) for 30 min, followed by two washing steps and resuspension in fresh RO medium. After 24 h of incubation, the lysate was centrifuged at 10 000 × *g* for 20 min and filtered through 0.22-μm filters (Millipore, MA, USA). Phage particles were precipitated overnight at 4°C with polyethylene glycol 8000 (final concentration, 100 g·L^−1^), collected by centrifugation at 10 000 × *g* for 60 min, and resuspended in 6-ml storage media (SM) buffer (100 mM NaCl, 8 mM MgSO_4_, 50 mM Tris–HCl, 0.01% gelatin [pH 7.5]). Further purification was achieved through CsCl equilibrium gradient centrifugation (Beckman Coulter, CA, USA) at 200 000 × *g* for 24 h. The visible phage band was extracted and dialyzed using 30 kDa super-filters (Millipore, MA, USA).

### Transmission electron microscopy inspection

Twenty microliters of desalted phage solution were spotted onto 200-mesh carbon-coated grids for 30 min, negatively stained with 1% phosphotungstic acid for 1 min, and air-dried for 30 min. Grids were examined using a JEM-2100 transmission electron microscope (JEOL, Tokyo, Japan) operating at 80 kV. Digital images were captured using the CCD image transmission system (Gatan Inc., CA, USA).

### Determining lytic profiles of inducible citromicrobial prophages

The lytic profiles of induced phages were determined using a spotting assay. Overnight bacterial cultures (1 ml) were combined with 5 ml of molten 0.5% soft agar and overlaid onto 1.5% bottom agar plates. After solidification, 5 μl aliquots of phage solutions were spotted onto bacterial lawns, with equivalent volumes of SM buffer serving as negative controls. Plaque formation was monitored over 24–48 h. For citromicrobial polylysogens containing multiple prophages, mono-lysogenic derivatives were first generated in the JL1366 background. These established mono-lysogens were then induced using MmC to produce purified phage stocks for host-range profiling.

### Construction of prophage-carrying strains

The prophage-free strain JL1366 was chosen as a baseline due to its susceptibility to most phage infections. Lysogenic mutants were screened out from viable colonies within lytic phage plaques following exposure to induced phage solution. Specifically, an individual plaque was removed and soaked in 1 ml phosphate buffered saline (PBS). After centrifugation at 7500 × *g* for 10 min, pelleted cells were washed twice with PBS to remove extracellular phage particles, and subsequently resuspended in 1 ml fresh RO medium. Serial dilutions (100 μl of 1100 cell suspensions) were plated on 1.5% RO agar plates and incubated at 28°C. Growing colonies were screened for lysogeny via colony PCR using genus-specific primers (Supplementary Table 2). Potential lysogens underwent three successive transfers to verify prophage integration stability, with lysogenic establishment ultimately confirmed by whole-genome sequencing.

### DNA sequencing and relative abundance analysis of induced citromicrobial prophages

The desalted phage solution was subjected to digestion with Protease K (100 mg·ml^−1^), sodium dodecyl sulfate (SDS; 10% [wt/vol]), and EDTA (0.5 mol·L^−1^; pH 8.0) at 55°C for 3 h. Following digestion, the solution was purified through sequential washes with phenol:chloroform:isoamyl alcohol (25:24:1 [w/v]) and chloroform:isoamyl alcohol (24:1 [w/v]). Phage DNA was then precipitated from the supernatant using isopropanol, and the resulting pellet was washed twice with 70% ethanol and air-dried. The purified DNA was resuspended in sterile Tris-EDTA buffer (10 mM Tris–HCl and 1 mM EDTA [pH 8.0]).

Sequencing was performed on the Illumina MiSeq platform with a PE300 DNA library, and clean reads were assembled de novo using Newbler v2.8 [43]. To analyze the relative abundance of different phages induced from citromicrobial polylysogens, reads aligning to each prophage genome were quantified using coverm (https://github.com/wwood/CoverM) and normalized by the respective prophage genome length. These normalized values were then used to calculate the relative abundance of each phage.

### Analysis of the relative Mu-type transposition target preference

Since each Mu-like genome is packaged along with flanking host sequences (5’flaps) from a different site in the bacterial chromosome, these 5’flaps are unique in every different phage capsid [44] and offer valuable information on transposition target sites. Sequencing reads of MmC-induced Mu-like phages were therefore employed in this analysis. The 5’flaps attached to both ends of the original prophage and its duplicated transposons were designated here as original and new 5’flaps, respectively. First, the 20-nt new 5’flaps attached to left and right ends of Mu-like genome were extracted, retaining only those sequences that perfectly mapped to the host chromosome (100% identity and query coverage in BLASTn analysis). The resulting genomic positions of qualified 20-nt new 5’flaps were visualized through BRIG [24] to show the chromosomal distribution pattern of Mu-like replicative transposons. These new 5’flap sequences were further aligned using the SeqLogo plug-in in TBtools [45] to identify 5-bp Mu-type target sequence consensus.

The high-coverage sequencing data of Mu-like phages in this study yielded sufficient number of new 5′-flaps (φA1, n = 54 135; φC, n = 32 187) for robust computational estimation of relative Mu-type transposition target preference (TTP). For each chromosomal gene, the TTP score was calculated by normalizing the count of 20-nt new 5’flaps matches against the frequency of 5-bp Mu-type target sequence consensus (NYSRN). Chromosomal genes were then ranked by their normalized TTP values and categorized into six bins. Statistical evaluation of subsystem category [46] enrichment or underrepresentation employed hypergeometric distribution probability (*P* < .01). Hot spots of Mu-type replicative transposition should be significantly enriched in top-ranking bins while being underrepresented in lower bins, whereas cold spots showed the opposite distribution pattern.

### *In silico* simulation of average burst size for Mu-like phages

The calculation was based on the biological principle [44] that, (i) each mature Mu-like phage particle corresponds to one single transposon copy present in the host cell at the end of the lytic cycle, and (ii) total transposons load per host cell comprise one original copy plus its duplicated transposons (*n*). If we count the original copy as *x* and its duplicated transposons as *y*, the average burst size would equal 1 + *y*/*x* (equivalent to 1 + *n*). When analyzing sequencing reads of Mu-like phages, we counted 20-nt original 5’ flaps as proxy for *x* and 20-nt new 5’flaps as proxy for *y*. Consequently, the average burst size was calculated by dividing the total number of original and new 5’flaps by the count of original 5’flaps.

### Detection of spontaneous prophage induction in citromicrobial lysogens

Cellular DNA was extracted from overnight cultures of citromicrobial lysogens and subjected to short-read sequencing. The identification of 20-nt new 5’flaps attached to left and right ends of the Mu-like genome (*L_NEW_* and *R_NEW_*) provided direct evidence of Mu-type prophage induction through replicative transposition events.

For integrase-encoding prophages (φD/φE), induction triggers homologous recombination between left and right prophage–bacterium junctions (*att*L and *att*R), resulting in phage attachment site (*att*P) restoration that links prophage genomic termini. Terminal redundancy analysis [47] revealed both φD and φE genomes possess *cos* sites (φD: 5’-GGC GTG GCG TGG GGG CGA G-3′; φE: 5’-CTA CGC CCC AC-3′), indicating their similar replication mechanism. Following prophage excision and circularization, rolling circle replication generates linear concatemeric phage DNA, with each progeny receives a genome cut at *cos* site. Consequently, the detection of 20-nt fragments spanning *att*P and adjacent prophage termini (*L-attP-R*) served as a molecular signature of prophage induction.

## Results and discussion

### Prophages shape bacterial microdiversity

**Figure 1:**
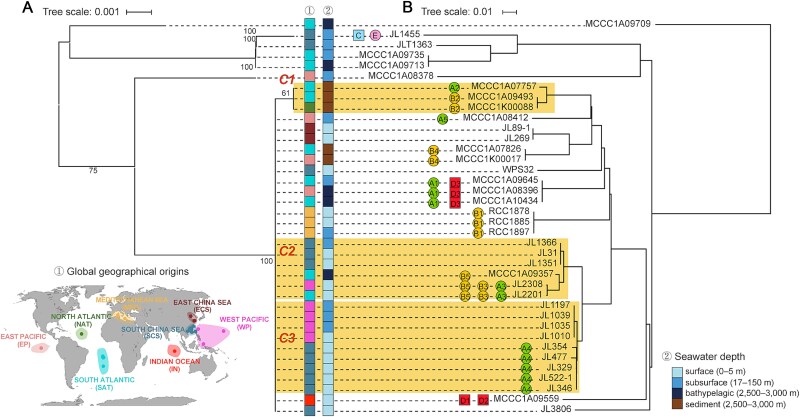
Phylogenetic analysis of 38 citromicrobial strains based on aligned 16S rRNA gene sequences (A) and whole genomes (B). Citromicrobial clonal clusters (C1–C3) were highlighted with orange backgrounds. Metadata describing the geographic origin and corresponding seawater depth for each citromicrobial strain was provided. Prophages were denoted by circular (siphophages) or rectangle (myophages) symbols, and were color-coded according to phage genus-level classification, with their genotypes annotated.

This study collected and sequenced 32 *C. bathyomarinum* strains and six *Citromicrobium* spp. strains originating from diverse oceanic regions. While most strains shared identical 16S rRNA gene sequences, substantial genomic diversity was observed at the whole-genome level (Fig. 1A, B). Pan-genome analysis of the 32 *C. bathyomarinum* genomes, which shared ANI of ≥95.5%, identified 6203 protein families, including 2291 core genes and 158 soft-core genes (Supplementary Fig. 1). Notably, prophage-encoded genes accounted for ~9% of host accessory genome (343/3754), underscoring their importance in shaping host pan-genome evolution. Three clonal groups (C1–C3) were identified, with intra-group members exhibiting exceptionally high genetic similarity (ANI ≥99.5%; Supplementary Fig. 2) and minimal density of SNPs across their aligned core genomes (2.8–9.5 SNPs/Mb; Supplementary Table 3). Strikingly, the majority (6/8) of observed genetic variations among intra-group members of three clonal groups were associated with prophage-related genomic islands (Supplementary Fig. 3). These findings collectively establish prophages as key determinants of bacterial microdiversity in marine *Citromicrobium*.

### Prophages display high genomic diversity due to potential HGTs

Genomic analysis identified 22 citromicrobial strains as lysogens that harbor at least one prophage, and two of them (strains JL2308 and JL2201) accommodating up to three prophages. The occurrence of lysogens observed here (58%) exceeded typical rates for marine bacteria (40%–50%) [48]. This propensity for lysogeny within this bacterial specie was further supported by two characterized citromicrobial phages: vB_CbaS-RXM, which targeted host tRNA-Val-GAC gene during its genome integration [49, 50], and vB_Cib_ssDNA_P1, whose close relative appeared as a prophage fragment in *Novosphingobium tardaugens* [51].

**Figure 2:**
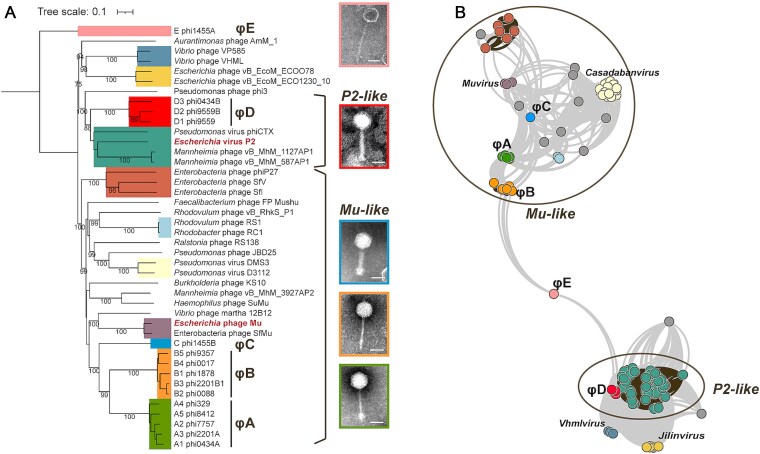
Evolutionary relationships among citromicrobial prophages and reference phage genomes. (A) Proteome-based phylogenetic analysis. The phylogeny was rooted at citromicrobial prophage φE, with bootstraps ≥75 shown. Branch annotations corresponds to viral clusters identified by vConTACT2. Citromicrobial prophage genus candidates were accompanied by representative electron micrographs, with a scale bar of 50 nm. Key phage groups, including P2-like phages (family *Peduoviridae*) and Mu-like phages, were labeled for reference. (B) Protein-sharing network analysis. Nodes represent individual phage genomes. Edges connecting phages within the same viral cluster were highlighted. The network was visualized using an edge-weighted, spring-embedded layout, with phage genomes sharing more protein clusters positioned in closer proximity.

Among 31 identified prophages (35 339–41 025 bp in lengths and 63.83%–66.37% in %G + C), we distinguished 15 genotypes that clustered into five candidate genera, designated φA to φE, through proteome-based comparative genomics (Fig. 2A, B). Approximately 50% of these prophages could be activated by MmC and produced phage particles, with transmission electron microscopy (TEM) imaging confirming siphophage/myophage morphologies (Supplementary Fig. 4). The φA, φB, and φC genera displayed Mu-like architectures, with φC largely conserving Mu-type head-myotail module, while φA and φB exhibited similar sipho-type tails–suggesting ancestral tail module recombination (Supplementary Fig. 5). Mu-type tail recombination has been documented before [10], our analysis further indicated Mu-type head recombination also occurred; φA retained a Mu-type head, whereas φB appeared to have acquired a novel head. Despite these variations, the φA, φB, and φC genera exhibited dual prophage/transposon nature, and we therefore proposed that these phages represented distinctive stages of Mu-type diversity continuum. The genus φD was recognized as P2-like (belonging to the *Peduoviridae* family), while φE represented an extremely unique chimera that combined tail-related and lysogeny-associated genetic features originated from φB and φD, respectively (Supplementary Fig. 5). These findings underscored the extensive genomic mosaicism shaped by recombination events in marine temperate phage populations.

### Two mechanisms for prophage integration and excision

**Figure 3:**
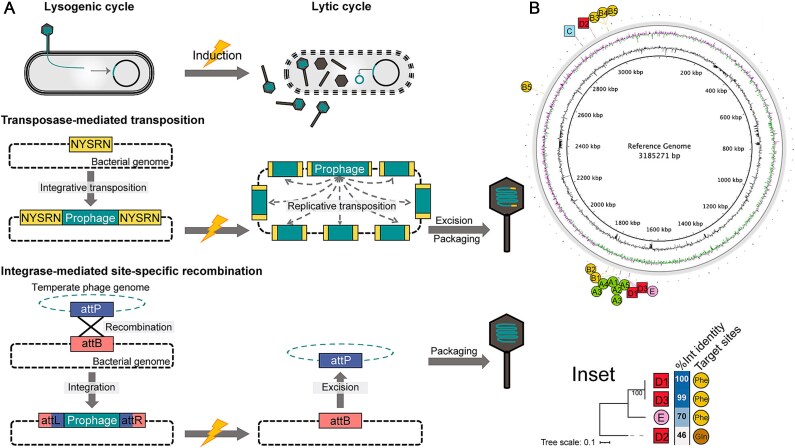
Integration and excision patterns of citromicrobial prophages. (A) Schematic diagrams illustrating transposase-mediated transposition and integrase-mediated site-specific recombination upon integration and induction. (B) Chromosomal integration sites mapped onto a reference genome derived from the fully sequenced JL2201 genome (GenBank CP155577.1) by removing all prophage genomes while preserving their natural attachment site in host chromosome (*att*B). Inset shows the neighbor-joining phylogeny of integrases from φD and φE, with prophage integrase sequence identity (based on BLASTp) and corresponding target sites indicated. Phe, tRNA-Phe-GAA; Gln, tRNA-Gln-TTG.

Whole-genome sequencing of MmC-induced phage particles (φA1, φB5, φC, φD3, and φE) enabled the comparison of prophage genomes with their encapsulated genomes, led to the identification of two pathways for prophage integration and excision (Fig. 3A).

Mu-like φA, φB, and φC genera employed transposase-mediated transposition. Following a Mu-type “nick-join-repair” pathway [52], duplicated 5-bp target host DNA with “NYSRN” (N/[T/C]/[G/C]/[G/A]/N) consensus [53, 54] were found at both ends of these prophage genomes (Supplementary Data Set 1). Nevertheless, they differ in target site bias during integrative transpositions (or integration). φA integrated into a 40-bp zone of a hypothetical host gene across all parental and newly lysogenized strains (n = 24), whereas φB showed moderate variation with six chromosomal hotspots identified from both parental and newly lysogenized strains (n = 20) (Fig. 3B and Supplementary Figs. 6, 7). These patterns differed notably from the prototype phage Mu, which is characterized by random DNA insertion during both integrative and replicative transpositions. During replicative transpositions (or induction), flanking host sequences (5’flaps) adjacent to both genomic ends of Mu-like prophages were encapsulated due to “head-full” packaging [55]. Since each Mu-like genome was packaged from a different site in the bacterial genome, the 5’flaps would be unique in every different phage capsid [44]. Analysis of host-derived 5’flaps present in sequencing data of phages φA1, φB5, and φC confirmed chromosome-wide distribution of replicative transposition sites (Supplementary Fig. 8), with significant correlation between “NYSRN” motif frequency per chromosomal gene and transposition incidence (Spearman *r*, 0.46; *P* < .0001; Supplementary Fig. 9).

φD and φE utilized integrase-mediated site-specific recombination, which requires precise alignment of attachment sites on phage and bacterial genomes (*att*P*–att*B pairing) for strand cleavage and exchange [50]. Phylogenetic analysis revealed the association between phage integrase and its adjacent *att*P sequence [56]: φD1, φD3, and φE shared closely related integrases and identical *att*P sites that target host tRNA-Phe-GAA gene, while φD2 possesses a less similar integrase and a distinct *att*P targeting host tRNA-Gln-TTG gene (Fig. 3 inset). This modularity explained how φD1 and φD2 avoid superinfection exclusion [57] within a single host, and suggested horizontal transfer of *att*P-integrase modules could either (i) enable niche partitioning between similar phages, or (ii) introduce dissimilar prophages at shared loci, which may enhance bacterial adaptative potential within clonal cell population.

### Citromicrobial prophages provide indirectly support for the PtW model

To investigate whether citromicrobial prophages follow the PtW paradigm–specifically whether increased host abundance favors lysogeny–we analyzed paired virome and metagenome data from the Tara Oceans dataset to interpret correlations between viral abundance, VHR, and host abundance (Supplementary Data Set 2).

**Figure 4:**
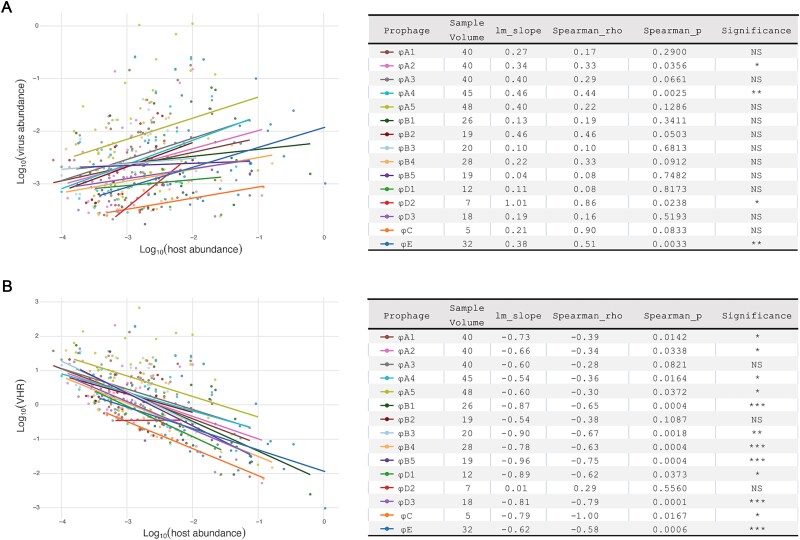
Correlations between virus abundances, VHR, and host abundance. (A) Log-transformed phage abundance (*y*) versus log-transformed host abundance (*x*). The relationship formula was *y* ~ *x*^lm_slope, where 0 < lm_slope < 1 and lm_slope = 1 indicated that *y* increased with *x* sublinearly and linearly, respectively. (B) Log-transformed VHR versus host abundance. Spearman’s correlation between the log–log data: NS, non-significant; ^*^P < .05; ^**^P < .01; ^***^P < .001.

The observed significantly sublinear (less than proportional; 0 < lm_slope < 1) growth patterns for φA2, φA4, and φE with increased host abundance, accompanied by strongly negative correlations between VHR and host abundance, align with PtW dynamics (Fig. 4A, B). A previous research by Alrasheed et al. suggested ecological interpretations of similar viral growth dynamics may include PtW dynamics, antagonistic virus–microbe dynamics, or trade-offs in Kill-the-Winner models [58]. Instead, φD2 displayed significantly linear growth pattern (lm_slope = 1) with evaluated host abundance and a weakly positive VHR–host abundance correlation, highlighting the intricate nature of prophage–host dynamics. While the well-defined prophage–host relationships in our study strengthen the validity of inferring phage strategies from metagenomic abundance correlations [59], we acknowledge the methodological constraints of this approach, particularly its inability to directly quantify lysogenic prevalence (or lytic activity) with increasing microbial abundances [58].

### Prophage-driven dual strategy: killing and colonizing the relatives

**Figure 5:**
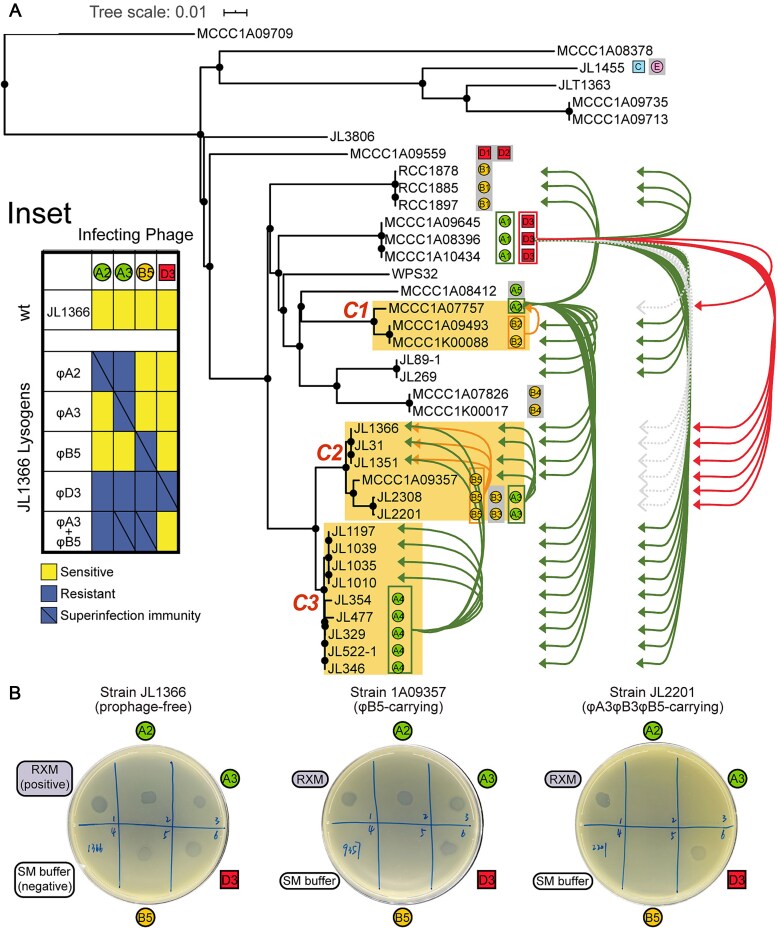
Phage sensitivity profiles of citromicrobial strains. (A) Lytic profiles of MmC-induced citromicrobial prophages. Each successful lysis was denoted by an arrow-headed line extending from the original prophage (or the parental host strain) to susceptible bacterial strains. The gray background behind the prophage icons highlighted those prophages that cannot be efficiently activated by MmC, potentially explaining their empty host-range profiles. (B) Phage spotting assay on bacterial lawns of three representative strains of clonal group C2. Blocks 1*–*6: 1, *C. bathyomarinum* phage vB_CbaS-RXM (positive control); 2, φA2; 3, φA3; 4, SM buffer (negative control); 5, φB5; 6, φD3. The vB_CbaS-RXM is a temperate phage isolated using JL1366 as a trapping host [49]. Since it can form plaques on all members of clonal group C2, it served as a positive control here. Inset showed phage sensitivity profiles of the baseline strain JL1366 (*wt*) and its derived lysogens against the phage collection. The double lysogen carrying both φA3 and φB5 was constructed to assess the additivity of resistance.

Cross-infection experiments revealed the phenomenon of superinfection immunity [42], that is, the lysogen is inherently resistant to its resident prophage. Meanwhile, the induced phages targeted the relative strain with a similar genetic background yet without the isogenic prophage, which thus lacking corresponding superinfection immunity (Fig. 5A). This “Kill-the-Relatives” phenomenon was particularly evident within clonal groups (C1–C3). Taking C2 as an example, φA3 lysed JL1366 and MCCC1A09357 but showed no lytic activity against its parental host JL2201. Similarly, φB5 lysed JL1366 while sparing its parental host MCCC1A09357/JL2201 (Fig. 5B).

The prophage-free strain JL1366 emerged as the most permissive host for phage replication, supporting plaque formation by multiple phages (φA2, φA3, φA4, φB5, and φD3). Using JL1366 as a baseline, viable colonies that were integrated by the prophage and had thus acquired immunity were isolated from phage plaques. The observed rates of lysogen were varied, such as 2.1% (φA2), 8.8% (φD3), 18.9% (φA3), and 20.8% (φB5) (Supplementary Figs. 10–13). These findings demonstrated that temperate phages employ a dual strategy of both eliminating (“Kill-the-Relatives”) and lysogenizing (“Colonize-the-Relatives”) genetically similar competitors. As a consequence, we procured five different JL1366-derived lysogens, with each harboring a unique prophage genotype. Phage sensitivity profiles of the wild-type JL1366 and its derived lysogens revealed a correlation between prophage carriage and phage resistance breadth (Fig. 5 inset). Compared with the mono-lysogens of φA3 and φB5 individually, a double lysogen harboring both φA3 and φB5 displayed resistance not only to its resident phages (φA3 and φB5) via superinfection exclusion but also to the non-resident phage φA2. Likewise, the triple lysogen JL2201, which harbors φA3, φB5, and φB3, showed additional resistance to φA2 (Fig. 5B). While the underlying mechanism requires further investigation, this enhanced resistance profile may stem from cooperative interactions between coexisting prophages.

Spontaneous prophage induction (SPI) was detected in overnight citromicrobial cultures (Supplementary Fig. 14), and the observed low-level phage release has been thought to be triggered by intrinsic cellular stress responses [42]. The capacity of spontaneous prophage activation suggests lysogeny represents a mutually beneficial strategy to some extent: prophages benefit from both lytic propagation (“Kill-the-Relatives”) and lysogenic colonization (“Colonize-the-Relatives”), and bacterial hosts acquire competitive advantages through phage resistance and elimination of susceptible competitors via SPI despite probabilistic host lysis [60]. It is worthy to point out that, while lysogeny occurs in only a minority (2.1%–20.8%) of post-infection viable cells, the “Colonize-the-Relatives” phenomenon introduces a strategic vulnerability (or a trade-off): new lysogens may acquire fitness through prophage carriage, potentially outcompeting the original lysogenic population that initiated the attack. This has been supported by a recent mathematic model [61], which predicts the optimal prophage fitness was to minimize lethality to the parental lysogen upon spontaneous induction while rapidly killing susceptible competitors and decreasing the formation of resistant lysogens.

### Mu-type within-host competitive advantage enlightened by transposition target preference

Polylysogeny, where multiple prophages coexisting within the same bacterial host [62], was prevalent in citromicrobial strain collection, which inevitably led to intense within-host phage competition. Sequencing data of MmC-induced phage particles from double lysogens MCCC1A10434 (φA1/φD3) and JL1455 (φC/φE) revealed disparities in sequencing coverage: Mu-like prophages dominated (φA1: 36236.24×; φC 39114.11×) over non-Mu-like partners (φD3: 21130.51×; φE: 568.96×), representing 63.1% (φA1) and 98.6% (φC) relative abundance respectively. This Mu-type competitive advantage was further qualitatively corroborated by TEM imaging (Supplementary Fig. 4) and aligned with their numerical dominance (25/31 prophages) and genetic diversity (11/15 genotypes) within the genus.

**Figure 6:**
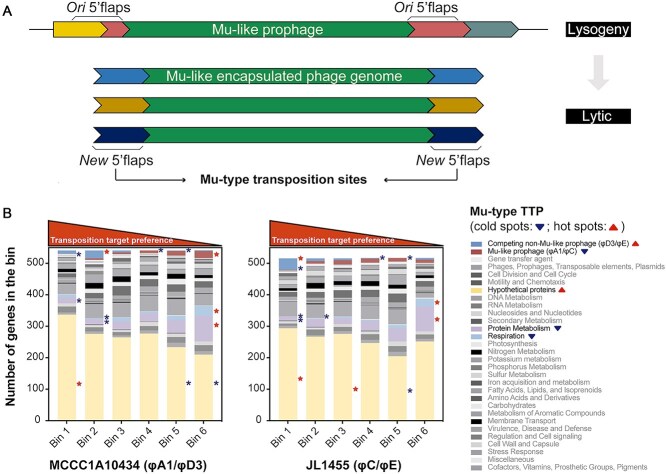
Relative Mu-type TTP. (A) Schematic illustration how to extract Mu-type transposition site information from the sequencing data of Mu-like phage particles. In Mu-like encapsulated genomes, the 5’flaps attached to both ends of the original prophage and its duplicated transposons were designated here as original and new 5’flaps, respectively. (B) Chromosomal genes of the two double lysogens, MCCC1A10434 and JL1455, were ranked based on their relative Mu-type TTP scores and categorized into six bins. Significant enrichment and underrepresentation (*P* < .01) for subsystem category were denoted with pentagrams and asterisks, respectively. Hot and cold spots for Mu-type replicative transposition were represented by triangles and inverted triangles, respectively.

Sequencing data of encapsulated genomes of Mu-like particles induced from the two double lysogens yielded interesting insights into the topic. By mapping new 5’flap sequences (Fig. 6A) across bacterial chromosomes (Supplementary Fig. 8), we quantified relative Mu-type TTP for each chromosomal gene by normalizing the count of 20-nt 5’flap matches against “NYSRN” motif frequency (Fig. 6B). Our data identified genes from competing non-Mu-like prophages and bacterial hypothetical genes as hot spots for replicative transpositions (*P* < .01). These observations align with prior work by Manna et al. examining Mu TTP in *Escherichia coli* and *Salmonella enterica* [63, 64], where they found defective *E. coli* prophages served as transposition cold spots while plaque-forming *S. enterica* prophages were moderate targets. Nevertheless, our analysis revealed no transposition bias when targeting different genes within competitor prophages, indicating uniform interference effects. Our extended examination of TTP across other phage-related elements reinforced the concept of selective targeting, with sporadic prophage-like genes and gene transfer agent (GTA)–like genes showing neutral TTP scores, indicating the lytic potential (virulence) of phage-like competitors may be a key determinant.

Significantly reduced transposition frequency (*P* < .01) was observed within two genomic regions: genes from Mu-like prophage self-regions and host genes related to respiration and protein metabolism. Considering average burst sizes of φA1 (106) and φC (82), the ratio of the length of Mu-like transposons to that of the host chromosome would be ~1:1 by the end of the lytic cycle. Successful phage production necessitates precise avoidance of self-transposition, mediated by previously characterized immune mechanisms [65]. The observed sparing of host genes for energy supply and protein biosynthesis reflects either their critical role in supporting the Mu-type lytic cycle or the lethal consequences of their disruption for phage progeny release. These findings illuminate a dual competitive strategy specific to Mu-like phages: (i) selective targeting of active coexisting prophages to monopolize host cell resources during lytic growth, and (ii) protective avoidance of self-damage and host metabolic genes critical for phage replication or release. Future efforts to assess Mu-type TTP across broader bacterial systems will be of great importance.

## Conclusions

Leveraging genomic, metagenomic, and experimental approaches, this study elucidates the profound impact of temperate phages on the ecology and evolution of marine citromicrobial host strains. Our findings established two critical phenomena requiring deeper mechanistic investigation: (i) the additive effect of phage resistance with higher prophage carriage, and (ii) the replicative interference between Mu-like prophages and co-resident prophages. Future investigations will employ integrated multi-omics methods to resolve the molecular determinants governing these intricate prophage–prophage and prophage–host interplays. This work provides a conceptual framework for understanding prophage-driven evolution in marine bacterial hosts, and we anticipate these insights can stimulate broader interests into prophage-host dynamics in marine ecosystems.

## Supplementary Material

FIG-S1_ycaf148

FIG-S2_ycaf148

FIG-S3_ycaf148

FIG-S4_ycaf148

FIG-S5_ycaf148

FIG-S6_ycaf148

FIG-S7_ycaf148

FIG-S8_ycaf148

FIG-S9_ycaf148

FIG-S10_ycaf148

FIG-S11_ycaf148

FIG-S12_ycaf148

FIG-S13_ycaf148

FIG-S14_ycaf148

Table-S1_ycaf148

Table-S2_ycaf148

Table-S3_ycaf148

Dataset_S1_ycaf148

Dataset_S2_VHR_ycaf148

## Data Availability

Citromicrobial host strains used in this study were obtained from either recognized culture collections or research collaborators: strains with prefixes “MCCC” are available from the Marine Culture Collection of China (https://www.mccc.org.cn/), “RCC” from the Roscoff Culture Collection (https://roscoff-culture-collection.org/), and “JL/WPS” strains can be provided upon request. Thirty-eight citromicrobial genomes have been deposited in the NCBI GenBank database, with their unique identifiers recorded in Supplementary Table 1. Comprehensive metadata for citromicrobial prophages, including precise genomic coordinates within bacterial chromosomes and *att*L/R junction annotations, are available in Supplementary Data Set 1. Complete genomes of the 15 citromicrobial prophage genotypes, specifically φA1–φA5, φB1–φB5, φC, φD1–φD3, and φE, can be conveniently downloaded from Figshare using the following doi: https://doi.org/10.6084/m9.figshare.25604454. Raw sequencing data are available under BioProject ID PRJNA1099946 (https://www.ncbi.nlm.nih.gov/bioproject/): (i) DNA of phage particles induced from MmC-treated lysogens, MCCC1A10434 (carrying φA1 and φD3), MCCC109357 (φB5-carrying), and JL1455 (carrying φC and φE); and (sii) cellular DNA from overnight cultures of two double lysogens, MCCC1A10434 and JL1455.
