# Supplementary material for: Prophages in marine Citromicrobium: diversity, activity, and interaction with the host
Source: ISME Commun. 2025 Aug 29;5(1):ycaf148. doi: 10.1093/ismeco/ycaf148 (PMC12486242; doi:10.1093/ismeco/ycaf148)
Supplement: FIG-S1_ycaf148 [file fig-s1_ycaf148.pdf]

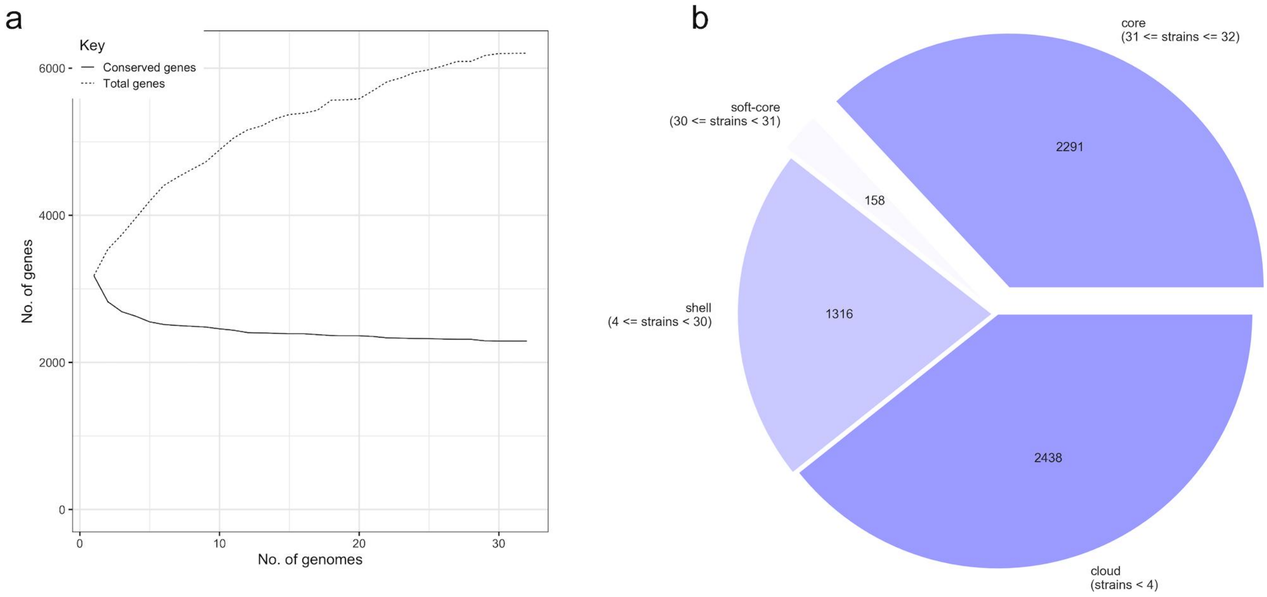

Fig. S1 The pan-genome calculated by 32 *C. bathyomarinum* genomes sharing pairwise ANI exceeding 95%. (a) The total numbers of genes from the pan-genome and core genome are plotted as a function of the number of genomes sequentially added. (b) The pan-genome pie chart.
