# Supplementary material for: Prophages in marine Citromicrobium: diversity, activity, and interaction with the host
Source: ISME Commun. 2025 Aug 29;5(1):ycaf148. doi: 10.1093/ismeco/ycaf148 (PMC12486242; doi:10.1093/ismeco/ycaf148)
Supplement: FIG-S2_ycaf148 [file fig-s2_ycaf148.pdf]

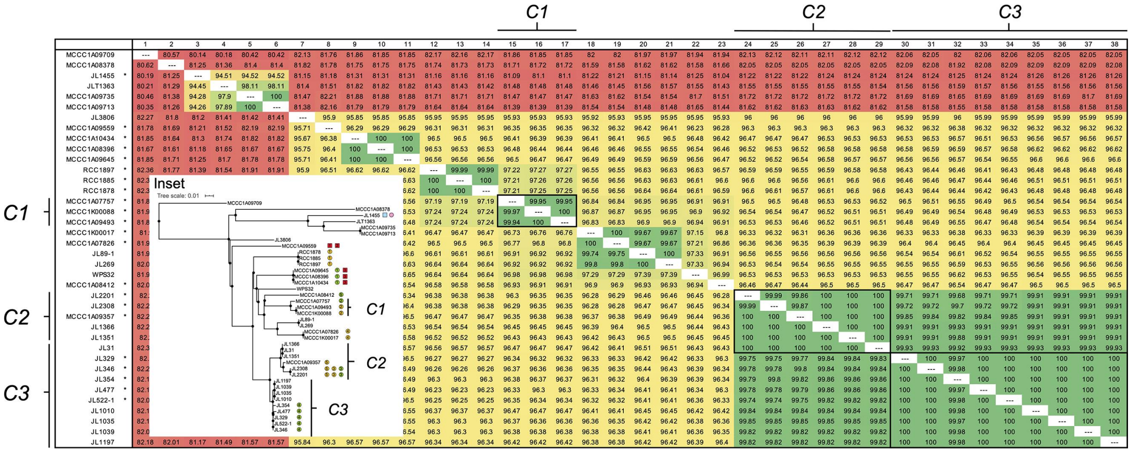

Fig. S2 Pairwise ANI of 38 citromicrobial genomes. The inset shows bacterial whole-genome phylogeny with symbols indicative of prophage genotypes. Clonal clusters C1–C3 are highlighted.
