# Supplementary material for: Prophages in marine Citromicrobium: diversity, activity, and interaction with the host
Source: ISME Commun. 2025 Aug 29;5(1):ycaf148. doi: 10.1093/ismeco/ycaf148 (PMC12486242; doi:10.1093/ismeco/ycaf148)
Supplement: FIG-S3_ycaf148 [file fig-s3_ycaf148.pdf]

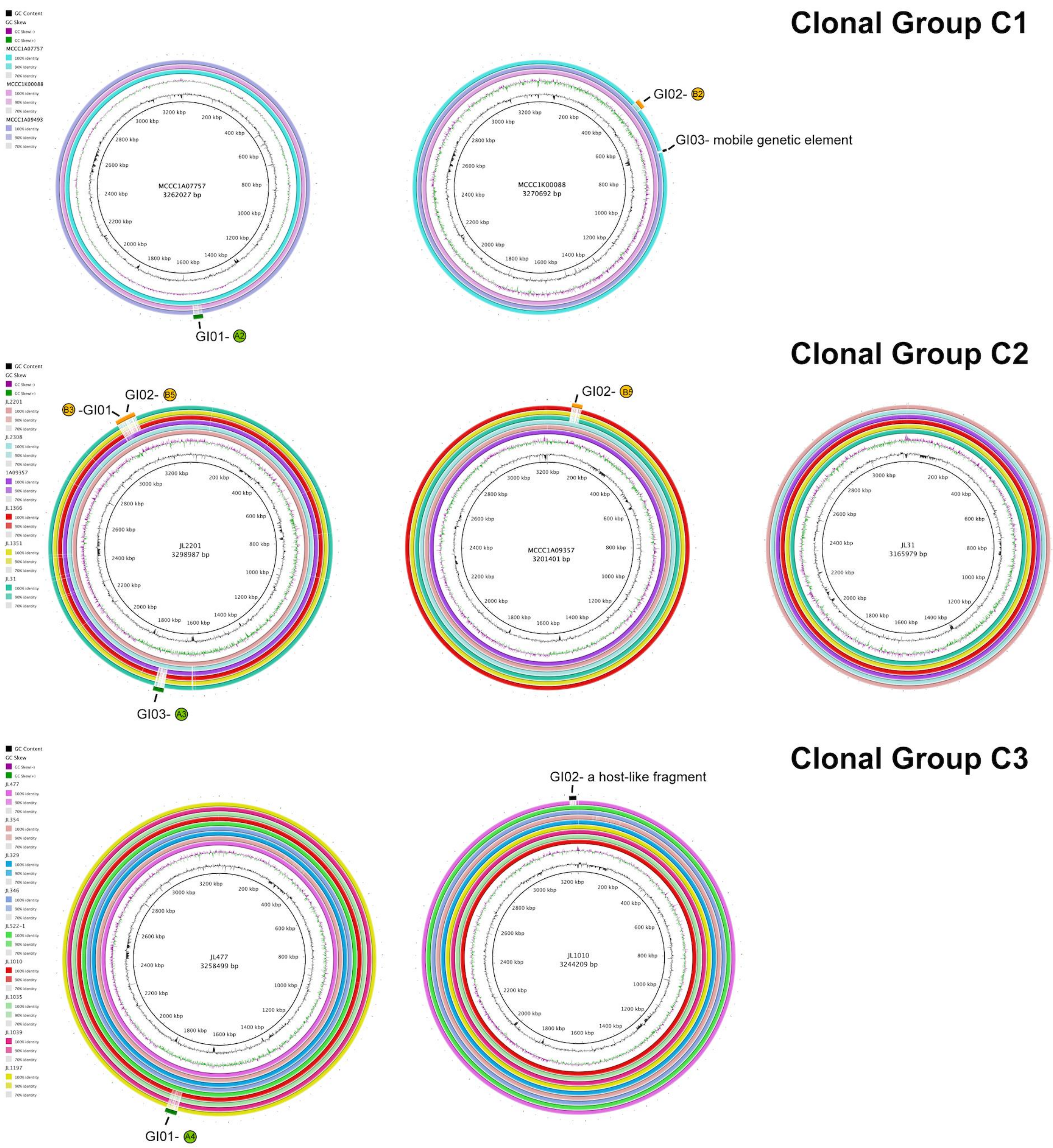

Inset

Clades	GIs	GI locus	GI length (bp)	GI content
C1	GI01	MCCC1A07757: 1278269–1316996 (contig2)	38,728	prophage φA2
	GI02	MCCC1K00088: 438483–476408 (contig1)	37,926	prophage φB2
	GI03	MCCC1K00088: 662867–672244 (contig1)	9,378	mobile genetic element
C2	GI01	JL2201: 3017019–3054843	37,825	prophage φB3
	GI02	JL2201: 3054980–3091987	37,008	prophage φB5
	GI03	JL2201: 1747309–1786176	38,868	prophage φA3
C3	GI01	JL477: 1769292–1808035	38,744	prophage φA4
	GI02	JL1010: 3224461–3237237 (contig1)	12,777	one host-like fragment

Fig. S3 Genetic islands (GIs) within clonal groups C1, C2, and C3. Chromosomal comparison of members within each clonal cluster. To present each GI, one representative of clonal bacterial genome takes a turn as the reference genome (the innermost ring). Inset table shows the content of GIs within each clonal cluster.
