# Supplementary material for: Prophages in marine Citromicrobium: diversity, activity, and interaction with the host
Source: ISME Commun. 2025 Aug 29;5(1):ycaf148. doi: 10.1093/ismeco/ycaf148 (PMC12486242; doi:10.1093/ismeco/ycaf148)
Supplement: FIG-S4_ycaf148 [file fig-s4_ycaf148.pdf]

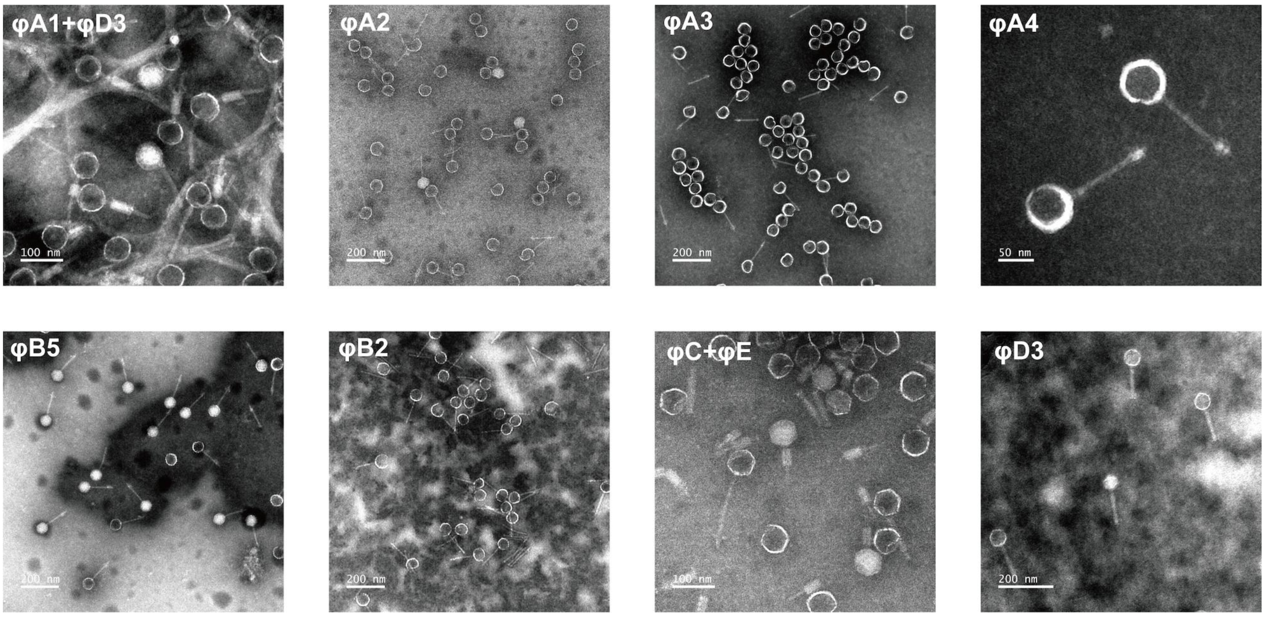

Fig. S4 TEM micrographs showing that several citromicrobial prophage genotypes, specifically φA1, φA2, φA3, φA4, φB2, φB5, φC, and φD3, could be substantially induced by Mitomycin C.
