# Supplementary material for: Prophages in marine Citromicrobium: diversity, activity, and interaction with the host
Source: ISME Commun. 2025 Aug 29;5(1):ycaf148. doi: 10.1093/ismeco/ycaf148 (PMC12486242; doi:10.1093/ismeco/ycaf148)
Supplement: FIG-S5_ycaf148 [file fig-s5_ycaf148.pdf]

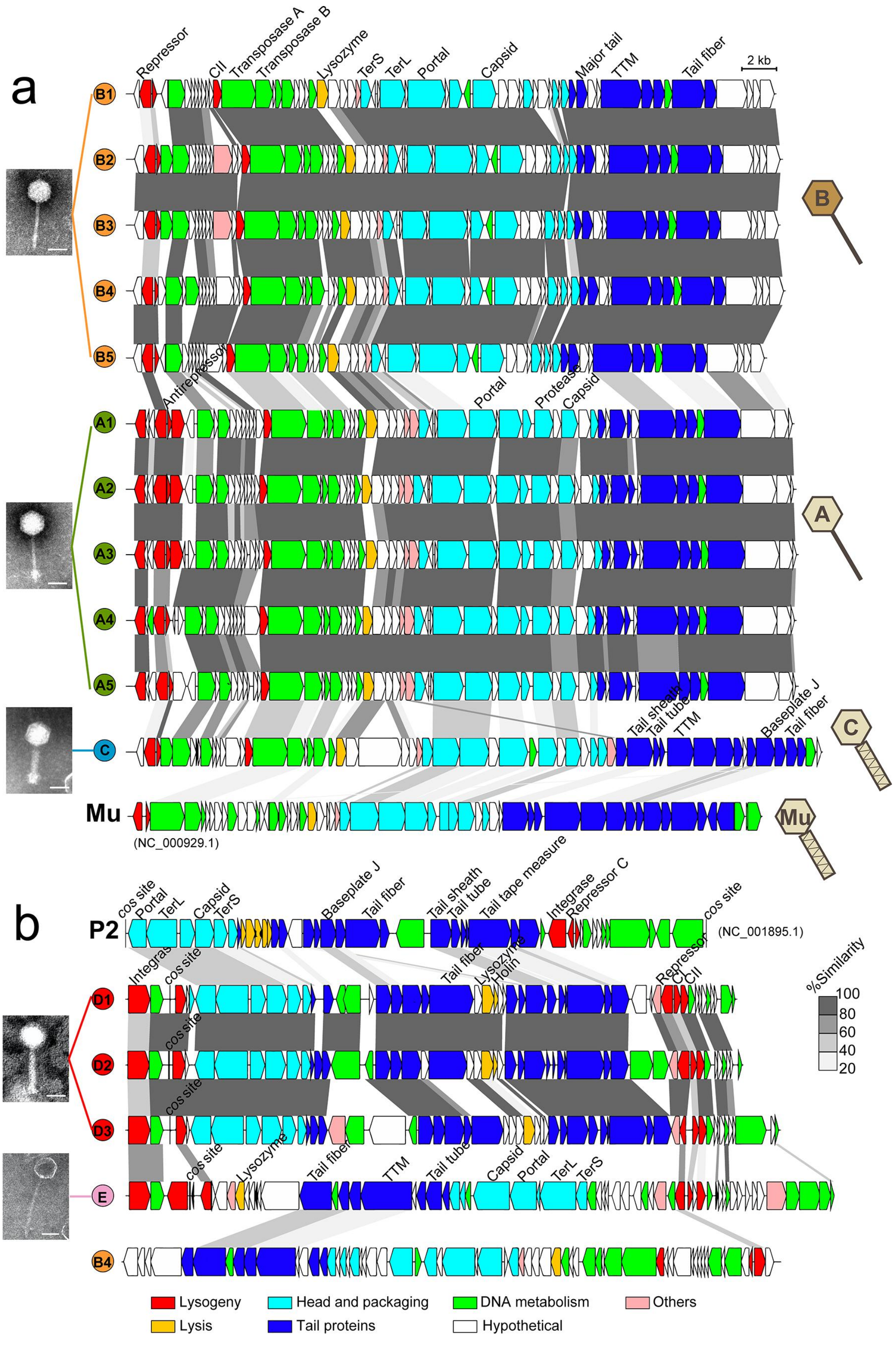

Fig. S5 Genetic and evolutionary traits of citromicrobial prophages. (a–b) Comparative genomic analysis of the 15 prophage genotypes and phage prototypes Mu (a) and P2 (b). Genes functional categories were color-coded (red, lysogeny-related; orange, lysis-related; cyan, head and packaging; blue, tail; green, DNA metabolism and transcription; white, hypothetical; pink, others). Sequence similarity of homologous gene pairs (based on BLASTp) is indicated by grayscale bars. Each candidate phage genus is accompanied by a representative electron micrograph, with a scale bar of 50 nm. Additionally, cartoon diagrams are included to illustrate distinctive stages of Mu-type diversity continuum.
