# Supplementary material for: Prophages in marine Citromicrobium: diversity, activity, and interaction with the host
Source: ISME Commun. 2025 Aug 29;5(1):ycaf148. doi: 10.1093/ismeco/ycaf148 (PMC12486242; doi:10.1093/ismeco/ycaf148)
Supplement: FIG-S6_ycaf148 [file fig-s6_ycaf148.pdf]

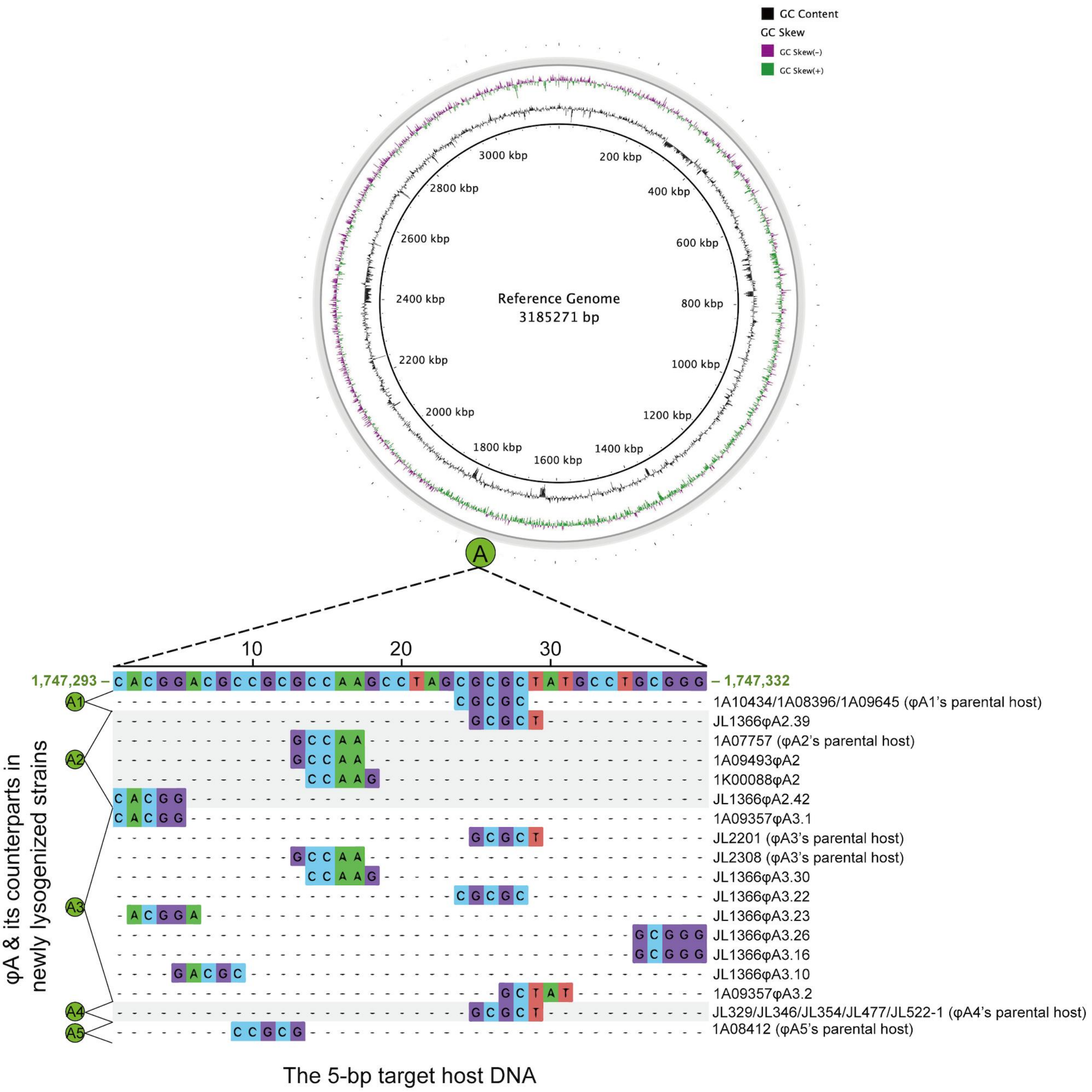

Fig. S6 Chromosomal sites of 5-bp target host DNA of φA and its counterparts in newly lysogenized citromicrobial strains. The reference genome is derived from a fully sequenced citromicrobial genome (GenBank CP155577.1) by removing all prophage genomes while preserving their natural attachment site in host chromosome (*att*B).
