# Supplementary material for: Prophages in marine Citromicrobium: diversity, activity, and interaction with the host
Source: ISME Commun. 2025 Aug 29;5(1):ycaf148. doi: 10.1093/ismeco/ycaf148 (PMC12486242; doi:10.1093/ismeco/ycaf148)
Supplement: FIG-S8_ycaf148 [file fig-s8_ycaf148.pdf]

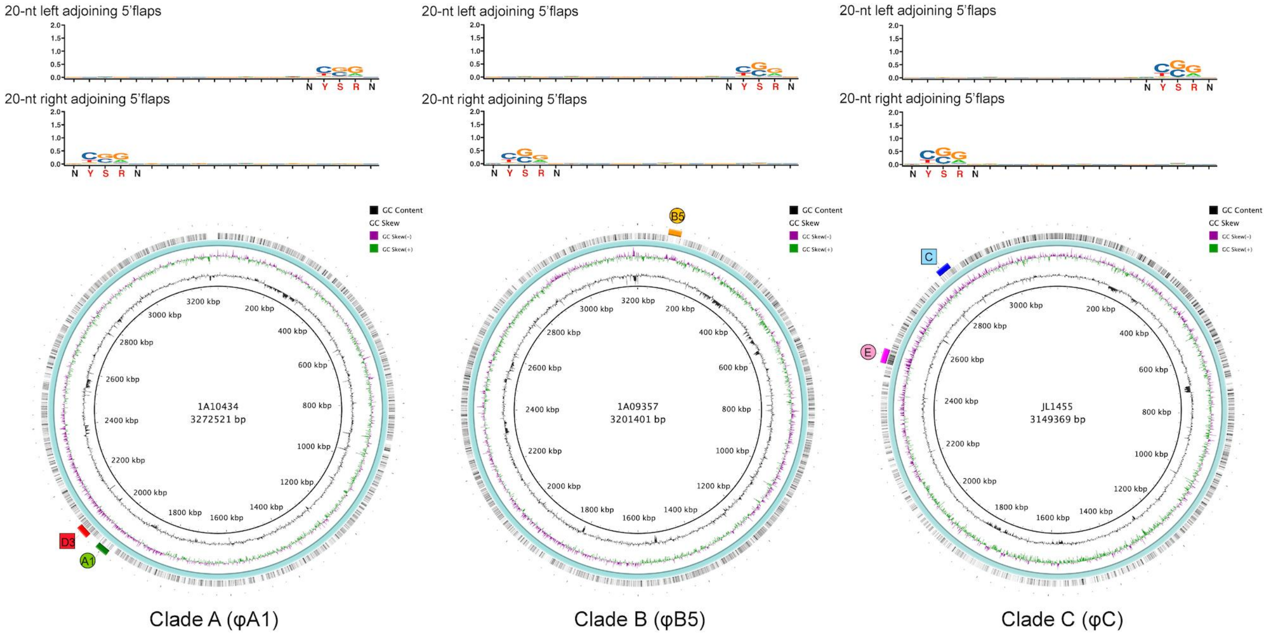

Fig. S8 Mu-type replicative transpositions. All 5'flaps flanking either Mu-like prophage end were extracted. The first 20 nt of 5'flaps were aligned to reveal the sequence pattern of 5-bp targeting sites of Mu-type replicative transpositions (the upper panel), and to understand their distribution along circular host chromosome (the lower panel).
