# Supplementary material for: Prophages in marine Citromicrobium: diversity, activity, and interaction with the host
Source: ISME Commun. 2025 Aug 29;5(1):ycaf148. doi: 10.1093/ismeco/ycaf148 (PMC12486242; doi:10.1093/ismeco/ycaf148)
Supplement: FIG-S9_ycaf148 [file fig-s9_ycaf148.pdf]

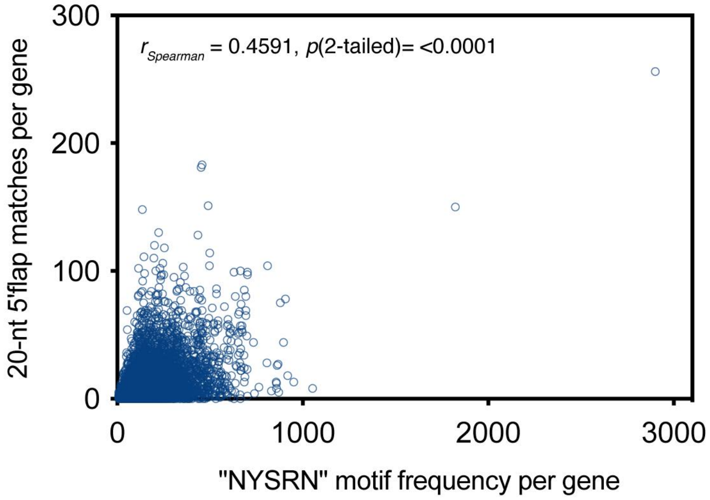

Fig. S9 Correlation between 20-nt 5'flap matches per chromosomal gene against its "NYSRN" motif frequency.
