# Supplementary material for: Prophages in marine Citromicrobium: diversity, activity, and interaction with the host
Source: ISME Commun. 2025 Aug 29;5(1):ycaf148. doi: 10.1093/ismeco/ycaf148 (PMC12486242; doi:10.1093/ismeco/ycaf148)
Supplement: FIG-S12_ycaf148 [file fig-s12_ycaf148.pdf]

%Incidence of Lysogen (φB5) = 10/48 = 20.8%

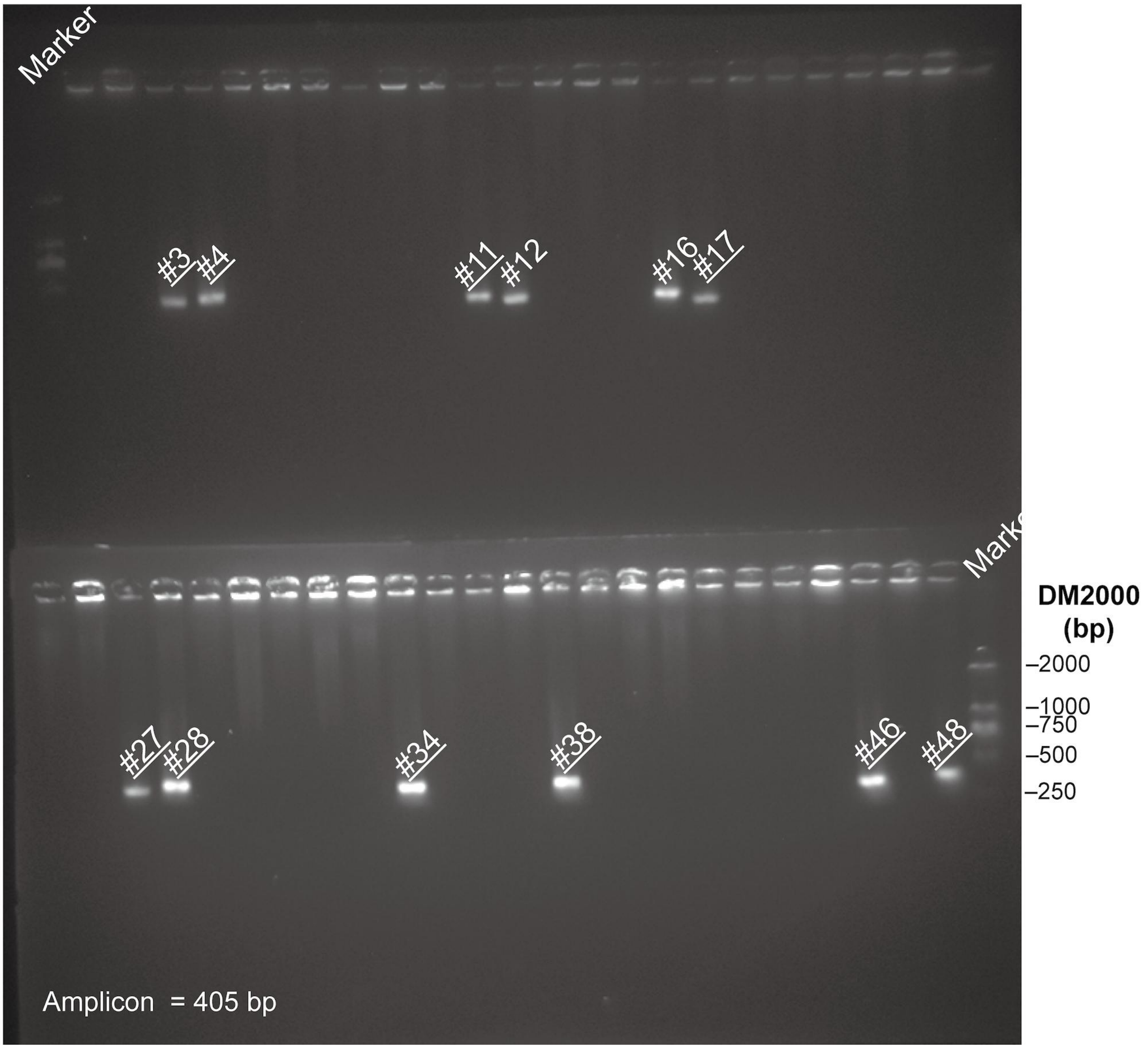

Fig. S12 JL1366-based colonies revived from φB5 phage plaque were screened for lysogeny using φB-specific primers. Putative lysogenized colonies were labeled with sequential numbers, with only those maintaining stable lysogeny through three consecutive transfers being underlined. The incidence of lysogen was determined by dividing the number of colonies with confirmed lysogenic establishment (validated by whole genome sequencing) by the total number of post-infection colonies tested.
