# Supplementary material for: Prophages in marine Citromicrobium: diversity, activity, and interaction with the host
Source: ISME Commun. 2025 Aug 29;5(1):ycaf148. doi: 10.1093/ismeco/ycaf148 (PMC12486242; doi:10.1093/ismeco/ycaf148)
Supplement: FIG-S14_ycaf148 [file fig-s14_ycaf148.pdf]

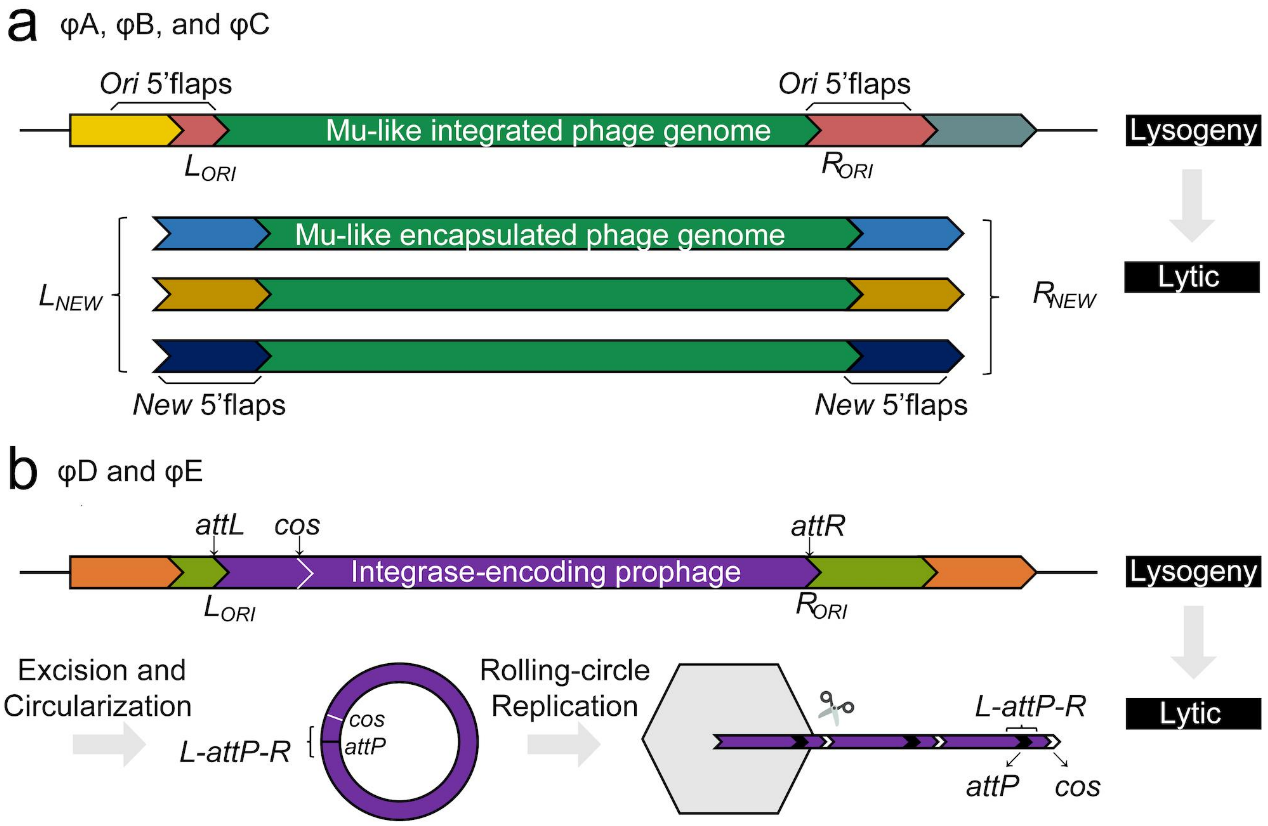

c

Lysogen	Prophage	#L_{ORI}	#R_{ORI}	#L_{NEW}	#R_{NEW}	#*L-attP-R*
MCCC 1A10434	φA1 (Mu-like)	158	128	14	7	*n/a*
	φD3 (P2-like)	201	156	*n/a*	*n/a*	5
JL1455	φC (Mu-like)	134	147	15	5	*n/a*
	φE	92	108	*n/a*	*n/a*	2

Fig. S14 Spontaneous prophage induction. Schematic illustration showing Mu-like genomes (a) and integrase-encoding genomes (b) under lysogeny and lytic cycles. (c) Sequencing reads from cellular DNA indicating spontaneous prophage induction. The identification of 20-nt new 5’flaps of left and right Mu-like ends (L_{NEW} and R_{NEW}) provided direct evidence of Mu-type prophage induction through replicative transposition events. Meanwhile, the detection of 20-nt fragments spanning *att*P and adjacent prophage termini (*L-attP-R*) served as a molecular signature of prophage induction of integrase-encoding phages.
