# Supplementary material for: Prophages in marine Citromicrobium: diversity, activity, and interaction with the host
Source: ISME Commun. 2025 Aug 29;5(1):ycaf148. doi: 10.1093/ismeco/ycaf148 (PMC12486242; doi:10.1093/ismeco/ycaf148)
Supplement: Table-S1_ycaf148 [file table-s1_ycaf148.pdf]

Table S1. Geographical information about the 38 citromicrobial isolates.

Strains[a,b]	Latitude (°)	Longitude (°)	Sources[c]	Water depths (m)	GenBank Accession
JL31	24.4584	118.247	SCS	0	GCF 001306285.1
JL1351	17.9936	120.2867	SCS	5	GCF_001306275.1
JL2201	-26.018	-13.8568	SAT	0	CP155577.1
JL1366	17.9936	120.2867	SCS	50	GCA_038594705.1
JL2308	18.3924	161.3252	WP	0	GCA_038594655.1
MCCC1A08378	-3.1027	-102.5535	EP	25	GCF_038594605.1
MCCC1A08412	-3.1027	-102.5535	EP	70	GCA_038594555.1
MCCC1A09357	-14.0491	-14.3844	SAT	2,927	GCA_038594545.1
MCCC1A09559	-6.9878	88.9906	IN	0	CP155064.1
MCCC1A09709	-15.2833	-13.6	SAT	2,700	GCF_038594485.1
JL354	21.6837	112.9182	SCS	0	GCF_000176355.1
JL477	22.1667	115.1528	SCS	0	GCF_001304795.1
WPS32	17	115	SCS	0	GCF_001306295.1
JLT1363	17.9936	120.2867	SCS	50	GCF_000186705.1
JL89-1	31.5082	122.9288	ECS	0	GCA_038594885.1
JL269	31.5	122.18	ECS	0	GCA_038594865.1
JL329	18.463	113.4912	SCS	0	GCA_038594895.1
JL346	20.7533	115.2422	SCS	0	GCA_038594825.1
JL522-1	22.2006	113.8024	SCS	0	GCA_038594835.1
JL1010	21	125	WP	0	GCA_038594795.1
JL1035	2	130	WP	50	GCA_038594755.1
JL1039	2	130	WP	50	GCA_038594735.1
JL1197	2	130	WP	50	GCA_038594775.1
JL1455	18.285	109.1072	SCS	17	GCF_038594725.1
JL3806	37.2181	119.8468	SCS	0	GCA_038594665.1
MCCC1A07757	-26.0211	-13.8521	SAT	2,545	GCA_038594645.1
MCCC1A07826	-26.0189	-13.8537	SAT	2,562	GCA_038594625.1
MCCC1A08396	-3.1027	-102.5535	EP	2,742	GCA_038594585.1
MCCC1A09493	-13.3552	-14.3112	SAT	3,142	GCA_038594495.1
MCCC1A09645	-15.2833	-13.6	SAT	50	GCA_038594515.1
MCCC1A09713	-15.2667	-13.6	SAT	2,700	GCA_038594465.1

Table S1. *Cont.*

Strains[a,b]	Latitude (°)	Longitude (°)	Sources[c]	Water depths (m)	GenBank Accession
MCCC1A09735	-15.2833	-13.6	SAT	150	GCA 038594385.1
MCCC1A10434	-15.0333	-13.0556	SAT	2,891	GCA_038594405.1
MCCC1K00017	-3.1041	-102.5544	EP	3,394	GCA_038594395.1
MCCC1K00088	14.7506	-44.9784	NAT	2,990	GCA_038594445.1
RCC1878	34.133	18.45	MED	5	GCF_001306355.1
RCC1885	34.133	18.45	MED	5	GCF_001306365.1
RCC1897	38.633	7.917	MED	85	GCF_001648095.1

[a]Strains bearing the prefixes "MCCC" and "RCC" were deposited in the Marine Culture Collection of China (MCCC) and the Roscoff Culture Collection (RCC), respectively;
[b]MCCC1A09709, MCCC1A09735, MCCC1A09713, MCCC1A08378, JL1455, and JLT1363 were taxonomically assigned as *Citromicrobium* spp., while the remainings belong to the species *Citromicrobium bathyomarinum*.
[c]SCS: South China Sea; SAT: South Atlantic Ocean; WP: West Pacific; EP: East Pacific; IN: Indian Ocean; ECS: East China Sea; NAT: North Atlantic Ocean; MED: Mediterranean Sea.
