# Supplementary material for: Prophages in marine Citromicrobium: diversity, activity, and interaction with the host
Source: ISME Commun. 2025 Aug 29;5(1):ycaf148. doi: 10.1093/ismeco/ycaf148 (PMC12486242; doi:10.1093/ismeco/ycaf148)
Supplement: Table-S2_ycaf148 [file table-s2_ycaf148.pdf]

Table S2. Primer pairs specific for citromicrobial prophages.

Primer name	Primer sequences	Targeted gene product	Amplicon size (bp)
φA_F	5'-ATA TCG ACG GGC ACA CCT TC-3'	Transposase A	703
φA_R	5'-TAG CGG TTG CCG TAG AGA TG-3'		
φB_F	5'-CGC AAG CTG TTC GGT TAT CG-3'	Transposase B	405
φB_R	5'-GAT CGC CTC GAT CGA AAG GT-3'		
φD3_F	5'-TCT CGG AGG AAA ATG TGG GC-3'	DUF4238	220
φD3_R	5'-GCG GTA TTA TGA GGG CTG GC-3'		
