# Supplementary material for: Prophages in marine Citromicrobium: diversity, activity, and interaction with the host
Source: ISME Commun. 2025 Aug 29;5(1):ycaf148. doi: 10.1093/ismeco/ycaf148 (PMC12486242; doi:10.1093/ismeco/ycaf148)
Supplement: Table-S3_ycaf148 [file table-s3_ycaf148.pdf]

Table S3. The SNP density of citromicrobial clonal groups.

**Clonal_Group**	**Number_of_ Genomes**	**Average_genome_length/bp**	**Core_genome_length/bp**	**SNPs**	**SNPs/Mb**
C1	3	3,267,833	2,951,493	23	7.8
C2	6	3,211,927	2,884,270	8	2.8
C3	9	3,261,904	2,932,041	28	9.5
C2&C3	15	3,241,913	2,779,518	111	39.9
